# Hereditary angioedema with an acute attack resolved after bone marrow transplantation for acute myeloid leukemia: a case report

**DOI:** 10.1186/s13223-023-00803-5

**Published:** 2023-05-16

**Authors:** Daisuke Honda, Isao Ohsawa, Masashi Aizawa, Yasuhiko Tomino, Katsuhiko Asanuma

**Affiliations:** 1 Department of Nephrology, Chiba University Hospital, Chiba, Japan; 2 Nephrology Unit, Internal Medicine, Saiyu Soka Hospital, Saitama, Japan; 3 Medical Corporation SHOWAKAI, Tokyo, Japan

**Keywords:** Acute attack, Bone marrow transplantation, C1-inhibitor, Hereditary angioedema, Japan, Leukemia

## Abstract

**Background:**

Hereditary angioedema (HAE), which is caused by C1-inhibitor (C1-INH) deficiency or dysfunction, is a rare and potentially life-threatening disease. In patients with HAE, excess production of bradykinin causes acute unpredictable recurrent attacks of angioedema in localized regions, including the larynx and intestines. Given the fact that HAE is an autosomal dominant disease, C1-INH produced in patients with HAE is 50% of that produced in healthy individuals. However, most patients with HAE present plasma C1-INH function of < 25% owing to the chronic consumption of C1-INH by kallikrein–kinin, contact, complement, coagulation, and fibrinolysis cascades. Recently, several therapeutic options have been developed for acute attacks and prophylaxis in the treatment of HAE; however, currently, there is no curative therapy for HAE.

**Case presentation:**

Here we report the case of a 48-year-old male patient who presented with a long-standing history of HAE and underwent bone marrow transplantation (BMT) for acute myeloid leukemia (AML) at the age of 39 years and has been in complete remission of AML and HAE thereafter. Notably, after BMT, his C1-INH function gradually increased as follows: < 25%, 29%, 37%, and 45.6%. Since his 20 s, he intermittently presented with an acute attack of HAE once every 3 months from the initial attack. Further, after undergoing BMT, the number of acute attacks decreased to twice within 4 years until the age of 45 years, and subsequently, the patient has been free of acute attacks. C1-INH is mainly synthesized by hepatocytes, but it is known to be partially produced and secreted from peripheral blood monocytes, macrophages, endothelial cells, and fibroblasts. We speculate that the C1-INH function may be increased by extrahepatic production of C1-INH, possibly synthesized by differentiated cells derived from hematopoietic and mesenchymal stem cells after BMT.

**Conclusions:**

This case report supports efforts to focus on extrahepatic production of C1-INH in the next strategy of new treatment development for HAE.

## Background

Hereditary angioedema (HAE), which is primarily caused by C1-inhibitor (C1-INH) deficiency (type I) or dysfunction (type II), is a rare autosomal dominant disease. The deficiency or dysfunction of C1-INH can lead to a paroxysmal increase in the production of bradykinin—a vasoactive peptide that triggers acute and recurrent attacks of localized subcutaneous or submucosal angioedema [1]. The initial symptoms of HAE typically appear in childhood and adolescence, and in patients with HAE, an unpredictable attack is potentially life-threatening as untreatable upper airway edema may cause asphyxia [2, 3]. Therefore, HAE should be diagnosed and an acute attack should be treated as early as possible [4, 5].

For patients with HAE, antihistamines, adrenaline, or corticosteroids are ineffective because HAE is mediated by bradykinin rather than histamine. In Japan, recently, the therapeutic approaches for acute attacks (on-demand treatment) and prophylaxis in HAE have been developing rapidly. The administration of icatibant—a bradykinin B2 receptor antagonist—which inhibits the binding of bradykinin to the receptor, has been used as a portable subcutaneous on-demand treatment [6]. Further, the kallikrein inhibitors berotralstat (oral) and lanadelumab (subcutaneous) have been used as long-term prophylaxis to prevent the production of bradykinin from the high molecular weight kininogen [7, 8]. C1-INH concentrates were originally used as intravenous on-demand treatment and short-term prophylaxis before performing invasive medical or dental procedures in patients with HAE to prevent acute attacks, but they have also been approved for use as long-term prophylaxis for subcutaneous regular replacement therapy. This treatment strategy is based on previous reports that plasma C1-INH function of approximately 40% of normal can significantly reduce the occurrence of an acute attack [9].

Herein, we report the case of a patient with HAE who underwent bone marrow transplantation (BMT) for replication of blood cells in the patient and production of additional normal blood cells for acute myeloid leukemia (AML), which resulted in a gradual increase in C1-INH function over 40%, subsequently ameliorating an acute HAE attack.

## Case presentation

**Fig. 1:**
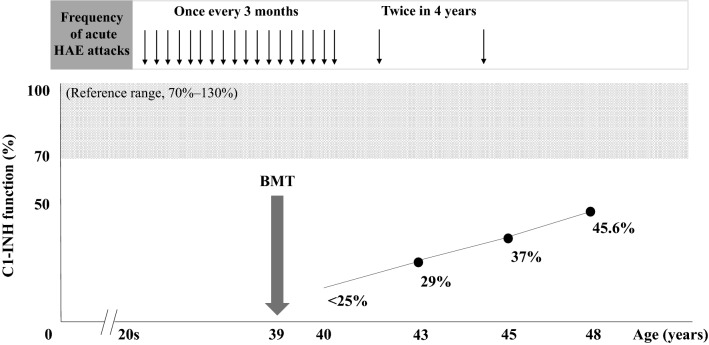
Clinical course of a patient with hereditary angioedema and acute myeloid leukemia. At the age of 39 years, the patient had AML and received BMT from a bone marrow bank. Consequently, he fully recovered from AML and remained in remission. Before he underwent BMT, he experienced acute HAE attacks approximately every 3 months from his 20 s. After BMT, his plasma C1-INH function increased as follows: < 25%, 29%, 37%, and 45.6%, and this increase in C1-INH function was associated with the reduced frequency of acute attacks. AML, acute myeloid leukemia; BMT, bone marrow transplantation; C1-INH, C1-inhibitor; HAE, hereditary angioedema

This report presents the case of a 48-year-old male patient with unexplained recurrent episodes of angioedema in the extremities and abdominal pain since his 20 s, lasting for several days and subsequently going into remission (Fig. 1). At the age of 39 years, the patient experienced AML (French–American–British subtype; M0) and received BMT from a bone marrow bank. Consequently, he completely recovered from AML and was in remission. Further, at the age of 40 years, he presented with facial and life-threatening laryngeal edema without any specific trigger, and his laboratory data revealed plasma C1-INH function of < 25% (reference range, 70–130%), serum C1-INH protein level of 7 mg/dL (reference range, 21–39 mg/dL), and serum C4 level of < 2 mg/dL (reference range, 14–36 mg/dL). Then, a repeat blood analysis was performed to confirm whether his laboratory findings were correct. Notably, his son also exhibited C1-INH function of < 20% and C4 level of 2.6 mg/dL. Accordingly, the patient met the criteria for diagnosis of HAE (type I) based on his clinical history, physical findings suggestive of acute attacks, the results of repeated complement studies, and a positive family history suggesting an autosomal dominant inheritance. Generally, genetic testing is not considered necessary when the clinical diagnosis is clear, as in the present case. Before undergoing BMT at the age of 39 years, our patient experienced acute HAE attacks approximately every 3 months, for which he had received C1-INH concentrates treatment, which was effective in alleviating symptoms. After BMT, his annual blood tests indicated that his C1-INH function gradually increased as follows: < 25%, 29%, 37%, and 45.6%. Furthermore, according to the increase in C1-INH function, the frequency of acute attacks decreased to twice in 4 years until the age of 45 years; finally, he has been free from acute attacks. Subsequently, with BMT, the patient has been in complete remission of both AML and HAE.

## Discussion and conclusions

Increased C1-INH function can prevent an acute attack in patients with HAE, for example, through a long-term prophylactic strategy of C1-INH concentrates replacement therapy, because an HAE attack is caused by excessive production of bradykinin due to C1-INH deficiency or dysfunction [9]. C1-INH produced in patients with HAE is 50% lower than that produced in healthy individuals because of an autosomal dominant inheritance caused by mutations in SERPING1. However, most patients with HAE exhibit C1-INH function of < 25% because of chronic consumption of C1-INH in the kallikrein–kinin, contact, complement, coagulation, and fibrinolysis cascades during homeostasis [10]. Therefore, replacement therapy with C1-INH concentrates is recommended as one of the first-line therapies for acute attacks, and for short- and long-term prophylaxes in the World Allergy Organization/European Academy of Allergy and Clinical Immunology (WAO/EAACI) guideline owing to the fact that C1-INH can help to comprehensively suppress several activations occurring throughout the abovementioned cascades, thereby inhibiting bradykinin production [11].

To the best of our knowledge, although no case of a patient with HAE receiving BMT has been reported, a patient with acquired angioedema (AAE) caused by acquired deficiency or dysfunction of C1-INH, receiving BMT, has been reported in a previous study [12]. AAE is considered to be caused by the activation of complement and consumption of C1-INH in lymphoproliferative and myeloproliferative diseases or by the development of autoantibodies to C1-INH. The prevalence of AAE is lower than that of HAE. According to the WAO/EAACI guideline, the clinical characteristics of AAE are late-onset angioedema (i.e., after the age of 30 years) in patients with no family history of angioedema and decreased serum C1q levels [11]. A 60-year-old male began suffering from angioedema attacks with decreased C1-INH function and was diagnosed with AAE owing to the late age of onset and a negative family history of angioedema. Further, he was treated with androgens, which reduced the frequency of attacks but did not render him completely free from attacks. At the age of 63 years, he was diagnosed with myelofibrosis and underwent BMT, which resulted in complete remission. Five months after BMT, his C1-INH function increased to the normal range, and 27 months later, he did not experience any acute attacks of angioedema. Therefore, it was considered that the improvement in AAE was caused by the complete remission of myelofibrosis by BMT. Meanwhile, in the present case, we considered that the patient had HAE and not AAE based on the earlier onset of angioedema (i.e., in his 20 s), a positive family history indicating autosomal dominant inheritance, and normal C1q level of 8.8 mg/dL (reference range, 8.8–15.3 mg/dL) at the age of 40 years.

Although C1-INH is mainly synthesized in hepatocytes, it is reported to be partially produced and secreted by peripheral blood monocytes, macrophages, endothelial cells, and fibroblasts [13–17]. In the present case, it was assumed that the gradual increase in C1-INH function up to 45.6% was due to the extrahepatic production of C1-INH synthesized from differentiated cells (such as monocytes, macrophages, endothelial cells, and fibroblasts) derived from hematopoietic and mesenchymal stem cells by BMT. Accordingly, we considered that C1-INH production by extrahepatic cells derived from BMT for AML contributed to the suppression of HAE attacks. The clinical course of our patient indicated that a single BMT may be as effective as regular replacement therapy with C1-INH concentrates for long-term prophylaxis. In the present case, the frequency of acute attacks was once every 3 months when C1-INH function was below 25%; however, when C1-INH function exceeded 25% after BMT and reached 37% at the age of 45 years, the attacks occurred twice in 4 years. Further, after the age of 45 years, attacks stopped to occur when C1-INH function exceeded 37%, and this finding is similar to that in a previous study [9].

In conclusion, to the best of our knowledge, this is the first study to report the case of a patient with HAE (type I) in whom angioedema resolved after BMT for AML with a corresponding increase in C1-INH function. This case report supports efforts to focus on extrahepatic production of C1-INH in the next strategy of new treatment development for HAE.

## Data Availability

The data that support the findings of this report are available from the corresponding author upon reasonable request.

## Abbreviations

AAE: Acquired angioedema
AML: Acute myeloid leukemia
BMT: Bone marrow transplantation
C1-INH: C1-inhibitor
HAE: Hereditary angioedema
WAO/EAACI: World Allergy Organization/European Academy of Allergy and Clinical Immunology
